# Substrate regulation on co-metabolic degradation of β-cypermethrin by *Bacillus licheniformis* B-1

**DOI:** 10.1186/s13568-019-0808-3

**Published:** 2019-06-12

**Authors:** Jiayuan Zhao, Dongying Jia, Juan Du, Yuanlong Chi, Kai Yao

**Affiliations:** 1 College of Light Industry, Textile & Food Engineering, Sichuan University, Chengdu, 610065 Sichuan People’s Republic of China; 2 College of Life Science, Sichuan Normal University, Chengdu, 610101 Sichuan People’s Republic of China; 3 The Faculty Geography Resource Science, Sichuan Normal University, Chengdu, 610101 Sichuan People’s Republic of China

**Keywords:** Beta-cypermethrin, Biodegradation, Co-metabolism, Substrate regulation, *Bacillus licheniformis* B-1

## Abstract

Beta-cypermethrin (β-CY) residues are a serious threat to food safety and human health. However, the residues are not efficiently biodegraded because microorganisms preferentially use the nutrients found in food and the environment for growth. In this study, the mechanisms underlying nutrient regulation during co-metabolic degradation of β-CY by *Bacillus licheniformis* B-1 were investigated. The strain B-1 resting cells and the suspension containing NaN_3_ showed no significant differences in β-CY degradation. The co-metabolic degradation and strain B-1 growth could be separately inhibited by iodoacetic acid and sodium fluoride. Adenosine monophosphate (AMP), fructose 1-6 bisphosphate (F1-6BP), Mg^2+^, and Mn^2+^ could improve the degradation, whereas adenosine triphosphate (ATP), alanine (Ala), phenylalanine (Phe), and phosphoenolpyruvate (PEP) were found to exert the opposite effect, indicating that β-CY degradation was positively associated with pyruvate kinase activity. Furthermore, glycerol, urea, ammonium chloride and peptone improved β-CY degradation in corn flour. The results provided a promising approach for nutrient regulation of pyrethroids biodegradation in food and the environment.

**Electronic supplementary material:**

The online version of this article (10.1186/s13568-019-0808-3) contains supplementary material, which is available to authorized users.

## Introduction

Various studies have focused on the degradation of organic contaminants by microorganisms because of advantages, including potential eco-friendly safety and practical applicability (Deng et al. 2015; Hussain et al. 2016; Tang et al. 2018; Zhao et al. 2015, 2016, 2018). Organic contaminants are degraded by microorganisms through co-metabolism, the primary mechanism for degradation, and mineralization (Luo et al. 2014; Tran et al. 2013). Many organic contaminants could be degraded by microorganisms via co-metabolism, but the degradation of some compounds was no efficient (Luo et al. 2014; Tran et al. 2013). Generally, co-metabolism is the process in which the obligatory presence of a growth substrate or another utilizable compound is critically needed to maintain biomass and induce the corresponding enzyme and/or cofactors for biodegradation (Tran et al. 2013). Therefore, the growth substrate is the key factor influencing co-metabolic degradation of organic contaminants by microorganisms.

Given that the use of organochlorine and organophosphorus pesticides has been totally or partly prohibited, pyrethroids have been widely used in agriculture and home formulations, accounting for about 25% of the worldwide pesticide market (Chen et al. 2013; Liu et al. 2014). Beta-cypermethrin (β-CY) is an important pyrethroid that accounts for more than 50% of the total production of pyrethroids market in China (Xiao et al. 2015). β-CY residues are organic contaminants that accumulate in human and animal bodies through the food supply chain, thereby causing toxic effects on the reproductive, immune, and nervous systems and gene expression (Jin et al. 2011; Mckinlay et al. 2008; Schettgen et al. 2002). To eliminate or reduce the levels of β-CY residues in food and the environment, a large number of microorganisms such as *Aspergillus niger* YAT, *Bacillus licheniformis* B-1, *Brevibacterium aureum*, and *Brevibacillus parabrevis* BCP-09, which could degrade it through co-metabolism have been screened and isolated (Chen et al. 2013; Deng et al. 2015; Liu et al. 2014; Tang et al. 2018). However, the degradation efficiencies by these microorganisms in the food and environment were limited. Substrate regulation might be a viable method to co-metabolic degradation of organic contaminants by microorganisms (Liu et al. 2013; Luo et al. 2014; Nzila 2013). Loh and Wang (1997) reported that sodium glutamate could promote the degradation of 4-chlorophenol by *Pseudomonas putida* ATCC 49451, glucose supplementation significantly reduced its degradation. However, few studies have focused on investigating the regulatory mechanisms and pathway for co-metabolic degradation of organic contaminants by microbial nutrients.

The efficiencies of co-metabolic degradation of organic contaminants by microorganisms are influenced by energy dependence on the degradation (Luo et al. 2014), the glucose metabolism pathway for providing cofactors of degradation (Liu et al. 2013), and the characteristics of degradation-associated enzyme or degrading enzyme (Tran et al. 2013). *Bacillus licheniformis* B-1 was isolated from soil of tea garden and found to degrade β-CY through co-metabolism (Liu et al. 2014; Zhao et al. 2015, 2016, 2018). In this study, the characteristics of β-CY degradation by strain B-1 was investigated, the regulatory mechanisms and pathway for the co-metabolic degradation was analyzed, and substrate regulation approaches of β-CY degradation were verified in corn flour. The objectives of this study were to clarify the mechanisms underlying nutrition regulation for co-metabolic degradation of organic contaminants by microorganisms and to develop an efficient method for microbial co-metabolic degradation of organic contaminants present in food and the environment.

## Materials and methods

### Materials

Beta-cypermethrin (99.7%) and acetonitrile of chromatographic grade were obtained from the National Standard Substances Center (Beijing, China) and Meridian Medical Technologies (Beijing, China), respectively. Acetonitrile, ethyl acetate, ethyl alcohol, iodoacetic acid, sodium fluoride, glucose, glycerol, urea, MgCl_2_, MnCl_2_, alanine (Ala), phenylalanine (Phe), and (NH_4_)_2_SO_4_ were of analytical grade and were procured from Kelong Chemical Co. (Chengdu, China). Nicotinamide adenine dinucleotide (NADH), adenosine monophosphate (AMP), fructose 1-6 bisphosphate (F1-6BP), adenosine triphosphate (ATP), and phosphoenolpyruvate (PEP) were purchased from Sigma.

### Microorganisms and media

The strain *Bacillus licheniformis* B-1, which is capable of degrading β-CY via co-metabolism was isolated from the soil in a tea garden (Ya’an, China) (Liu et al. 2014; Zhao et al. 2015, 2016, 2018). Based on the analysis of morphological, physiological, biochemical characteristics and 16S rDNA sequence (GenBank Accession No.: HQ009796), strain B-1 was identified as *Bacillus licheniformis* (Liu et al. 2014), which has been stored in China Center for Type Culture Collection (NO. CCTCC M 2016258). Strain B-1 was stored in 15% (v/v) glycerol solution at − 80 °C before experiments.

Luria–Bertani (LB) medium containing 5.0 g/L of yeast extract, 10.0 g/L of peptone and 10.0 g/L of NaCl was prepared. LB-CY medium consisted of LB medium and β-CY (100 mg/L). The pH value of each medium was adjusted to 7.0–7.5, and 0.2% (v/v) of ethyl alcohol was added into the medium as a hydrotropic agent before sterilization at 121 °C for 20 min (Liu et al. 2014; Zhao et al. 2015).

### Inoculum preparation

Strain B-1 was thawed and inoculated into a 100 mL Erlenmeyer flask, which contained 30 mL LB medium with 100 mg/L β-CY. Then the flask was placed in a rotary shaker (Multitron-Pro, Infors, Switzerland) at 180 rpm and 30 °C, and the activated strain was obtained at 16 h cultivation. After being centrifuged (ST40R, Thermo, German) at 10,000 rpm and 4 °C for 10 min, the strain was collected and suspended in sterile N-saline (0.9% NaCl) to achieve the cell density of about 1.0 × 10^8^ cells/mL. Then, the bacterial suspension was used as inoculum (Liu et al. 2014; Zhao et al. 2015). The resting cells of strain B-1 were obtained after allowing the inoculum to stand for 48 h at 30 °C (Sabullah et al. 2016).

### Determination of β-CY concentration

The β-CY concentration was determined as previously described (Liu et al. 2014; Zhao et al. 2015). In detail, 5 mL of LB-CY medium and acetonitrile were transferred into a 100 mL flask and shaken for 30 s by a vortex mixer. Then, the flask was under ultrasonic (40 kHz and 300 W) for 30 min (Liu et al. 2012). After the mixture was centrifuged at 8000 rpm for 20 min, the supernatant was collected and filtered through a 0.22 μm membrane filter. Finally, the obtained filtrate was used to determine its β-CY content by high-performance liquid chromatograph (HPLC). The content of β-CY was determined by LC-20AT HPLC (Shimadzu, Kyoto, Japan) equipped with LC-20AT pump (Shimadzu), a CTO-20A column oven (Shimadzu), a Kromasil C_18_ column (250 mm × 4.60 mm, 5.0 μm; Sweden) and an SPD-M20A detector. Degradation of β-CY was calculated according to the following equation:

1 $${\text{Degradation}}\left( \% \right) = \left( {1{-}{{\text{C}} / {{\text{C}}_{0} }}} \right) \times 100$$

where *C* and *C*_0_ are the concentration of β-CY (mg/L) in inoculated medium and the control, respectively.

### Energy dependence degradation assay

The growth substances metabolism provided energy or co-factors for microorganisms to degrade organic contaminants via co-metabolism (Luo et al. 2014; Nzila 2013; Tran et al. 2013). To elucidate the energy dependence in the co-metabolic degradation of β-CY, the degradation by resting cells of strain B-1, strain B-1 suspension, and strain B-1 suspension containing sodium azide (NaN_3_) were investigated. For the energy dependence degradation assay, three samples, namely, the suspension of strain B-1, the suspension of strain B-1 with the inhibitors of 30 mmol/L NaN_3_, and strain B-1 resting cells, were used to confirm whether the co-metabolic degradation of β-CY was energy-dependent (Hua et al. 2013; Sabullah et al. 2016). The initial β-CY concentration in all three samples was 30 mg/L. Samples were incubated at 180 rpm and 30 °C for 12 h and subsequently analyzed as described above.

### Effect of glucose metabolism inhibitors on co-metabolic degradation of β-CY by strain B-1

The energy or co-factors required by co-metabolic degradation of organic contaminants were from the microbial metabolism (Luo et al. 2014; Nzila 2013; Tran et al. 2013). Therefore, it is necessary to study the correlation between glucose metabolism and co-metabolic degradation of β-CY by strain B-1. Sodium fluoride, iodoacetic acid, trisodium phosphate and malonic acid were selected as the inhibitors of glucose metabolism (Shiota 1957; Wright 1937; Tang et al. 2011). Afterwards, a series of LB-CY media containing each of the inhibitors at 500 mg/mL were prepared. A 28.5 mL volume of LB-CY medium containing each inhibitor was mixed with 1.5 mL of inoculum in a 250 mL flask. The flask was shaken (180 rpm) at 30 °C for 72 h. The LB-CY medium without the inhibitor was used as control. Degradation of β-CY was calculated by Eq. (1), and the OD_600_ was determined as previously described (Liu et al. 2014; Zhao et al. 2015).

### Characteristics of co-metabolic degradation of β-CY by strain B-1

The co-factors or inhibitors involved in co-metabolism such as metallic ions, ATP, NADH, or some intermediated metabolites play an important role for β-CY degradation, and the effects of these compounds on the degradation is needed to further investigate. Strain B-1 suspensions containing 30 mg/L β-CY was used in the experiment. As shown in Table 1, MnCl_2_, MgCl_2_, ZnCl_2_, CaCl_2_, ATP, NADH, PEP, AMP, F1-6BP, AMP, Ala, and Phe were added to the mixture, and the samples were prepared. Some metallic ions were the key factors for the actions of inhibitors and activators during co-metabolic degradation of organic contaminants (Deng et al. 2015; Guo et al. 2009), and MgCl_2_ was used in the experiment for addition of inhibitors and activators. The sample was shaken (180 rpm) at 30 °C for 72 h. The residual β-CY concentration was measured by HPLC, and the Eq. (1) was used to compute the degradation of β-CY.

**Table 1:** Samples compositions with the different tested compounds

Sample	MgCl_2_ (mmol/L)	MnCl_2_ (mmol/L)	ZnCl_2_ (mmol/L)	CaCl_2_ (mmol/L)	ATP (mmol/L)	NADH (mmol/L)	PEP (mmol/L)	AMP (mmol/L)	F1-6BP (mmol/L)	Ala (mmol/L)	Phe (mmol/L)
Control	–^a^	–	–	–	–	–	–	–	–	–	–
1	0.1	–	–	–	–	–	–	–	–	–	–
2	0.5	–	–	–	–	–	–	–	–	–	–
3	1.0	–	–	–	–	–	–	–	–	–	–
4	2.0	–	–	–	–	–	–	–	–	–	–
5	–	0.1	–	–	–	–	–	–	–	–	–
6	–	0.5	–	–	–	–	–	–	–	–	–
7	–	1.0	–	–	–	–	–	–	–	–	–
8	–	2.0	–	–	–	–	–	–	–	–	–
9	–	–	0.1	–	–	–	–	–	–	–	–
10	–	–	0.5	–	–	–	–	–	–	–	–
11	–	–	1.0	–	–	–	–	–	–	–	–
12	–	–	2.0	–	–	–	–	–	–	–	–
13	–	–	–	0.1	–	–	–	–	–	–	–
14	–	–	–	0.5	–	–	–	–	–	–	–
15	–	–	–	1.0	–	–	–	–	–	–	–
16	–	–	–	2.0	–	–	–	–	–	–	–
17	–	–	–	–	5.0	–	–	–	–	–	–
18	–	–	–	–	–	5.0	–	–	–	–	–
19	–	–	–	–	–	–	5.0	–	–	–	–
20	–	–	–	–	–	–	10.0	–	–	–	–
21	–	–	–	–	–	–	15.0	–	–	–	–
22	–	–	–	–	–	–	20.0	–	–	–	–
23	–	–	–	–	–	–	–	5.0	–	–	–
24	–	–	–	–	–	–	–	–	5.0	–	–
25	0.1	–	–	–	–	–	–	–	5.0	–	–
26	0.1	–	–	–	–	–	–	5.0	–	–	–
27	0.1	–	–	–	–	–	–	–	–	5.0	–
28	0.1	–	–	–	–	–	–	–	–	–	5.0

^a^Not added

### Nutrient regulation of co-metabolic degradation of β-CY by strain B-1 in corn flour

Corn flours were purchased from Guo Jiaqiao market, Chengdu, Sichuan province, China. Corn flour (30 g) was mixed with 19.5 mL of purified water, and a series of mixtures with varying concentrations (2.0, 4.0, 6.0, 8.0 and 10.0 g/kg) of glucose, glycerol, urea, ammonium chloride and peptone were prepared, respectively. The resulting mixtures were sterilized at 121 °C for 20 min. Then, the β-CY and strain B-1 suspensions were added to the mixtures to achieve final concentrations of 50 mg β-CY per kg corn flours and 1.0 × 10^8^ cells per gram corn flours. Samples were inoculated at 180 rpm and 30 °C for 72 h. β-CY concentrations were measured following the methods of Liu et al. (2014) and Zhao et al. (2015), and the degradation was calculated.

### Statistical analysis

Each experiment was performed three times. Data were expressed as the means ± standard deviations of three replicates. All statistical analyses were performed using SPSS v 17.0 (SPSS Inc., Chicago, IL, USA).

## Results

### Energy dependence on the degradation of β-CY by strain B-1

Energy-containing molecules, such as ATP or guanosine triphosphate (GTP), could be produced in microbial suspension (Chauhan et al. 2003; Errécalde et al. 2004), but not in resting cells or strain B-1 suspension containing NaN_3_, in which energy production is exhausted or blocked by NaN_3_ via inhibition of oxidative phosphorylation (Chauhan et al. 2003; Errécalde et al. 2004). As shown in Table 2, the strain B-1 suspension, resting cells, and strain B-1 suspension containing NaN_3_ showed no significant differences in β-CY degradation. It indicated that β-CY degradation by strain B-1 did not require energy.

**Table 2:** Degradation of β-CY by resting cells and strain B-1 cells in suspension containing NaN_3_

Sample	β-CY degradation (%)
Strain B-1 suspension	23.42 ± 0.36^a^
Resting cell	23.35 ± 0.28^a^
Strain B-1 suspension with NaN_3_	22.55 ± 0.57^a^

Data are presented as mean ± standard deviation of three replicates; the standard deviations are within 5% of the mean. Different letters indicate significant differences (*p* < 0.05)

### Effect of glucose metabolism inhibitors on the co-metabolic degradation of β-CY by strain B-1

**Fig. 1:**
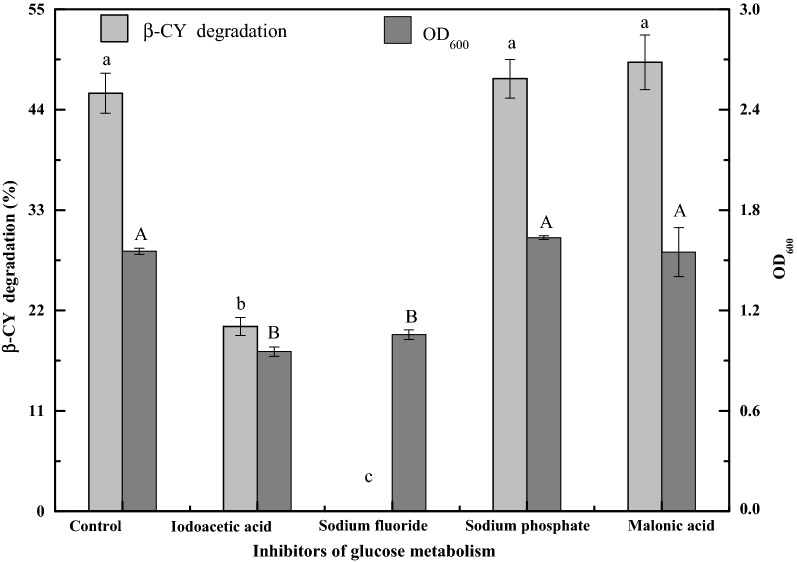
Effects of glucose metabolism inhibitors on co-metabolic degradation of β-CY by strain B-1. Data are presented as mean ± standard deviation of three replicates; the standard deviations are within 5% of the mean. Different letters indicate significant differences (*p* < 0.05)

Some studies demonstrated that Embden–Meyerhof–Parnas (EMP) pathway, hexose monophosphate pathway (HMP) and tricarboxylic acid (TCA) cycle are the primary pathways involved in the glucose metabolism in *Bacillus licheniformis* (Calik and Ozdamar 1998; Voigt et al. 2004). Therefore, further studies are required to investigate the relationships among these pathways in glucose metabolism and β-CY degradation in strain B-1. Some compounds, such as iodoacetic acid and sodium fluoride, which inhibit the EMP pathway, and trisodium orthophosphate and malonic acid, which inhibit the HMP and TCA cycle, respectively, can additionally inhibit glucose metabolism (Shiota 1957; Wright 1937; Tang et al. 2011). The effects of glucose metabolism inhibitors on co-metabolic degradation of β-CY by strain B-1 in LB-CY medium are presented in Fig. 1. The degradation of β-CY and the OD_600_ of strain B-1 in control samples were almost equal to those in the samples containing sodium phosphate and malonic acid, indicating that HMP and TCA cycle in strain B-1 were not correlated with the growth of cells and co-metabolic degradation of β-CY. Nevertheless, the degradation of β-CY in the samples containing iodoacetic acid and sodium fluoride significantly deceased to 20.25% (*p *< 0.05) and 0.15% (*p* < 0.01), respectively. The OD_600_ value of strain B-1 in these two samples decreased by 38.61% and 32.05% relative to the control, respectively. The above results demonstrated that co-metabolic degradation of β-CY was correlated with EMP of strain B-1 in LB medium.

### Characteristics of the co-metabolic degradation of β-CY by strain B-1

To investigate the characteristics of the organic contaminant degradation, the compounds involved in the HMP and TCA were used to regulate the degradation describing by Liu et al. (2013) and Lu et al. (2018), respectively. Some researches also indicated that the biodegradation of organic contaminants by microorganisms or the growth of cells could be significantly affected by adding the compounds required by the glucose metabolism pathway (Gong et al. 2008; Hwang et al. 2011; Ostengo et al. 2005). Therefore, the relationship between the EMP in strain B-1 and the co-metabolic degradation of β-CY was necessary to investigate, and the effects of specific compounds involved in the EMP on the co-metabolic degradation of β-CY were evaluated.

### Effects of divalent metal ions on the co-metabolic degradation of β-CY

The effects of Mg^2+^, Mn^2+^, Zn^2+^ and Ca^2+^ on the co-metabolic degradation of β-CY by strain B-1 suspension are shown in Table 3. Results indicated that Mg^2+^ and Mn^2+^ could improve the co-metabolic degradation of β-CY under optimal conditions. The degradation of β-CY was found to increase when the Mg^2+^ and Mn^2+^ concentrations were increased from 0.1 to 0.5 mmol/L. At cation concentrations higher than 0.5 mmol/L, β-CY degradation was found to decrease with increasing concentrations of Mg^2+^ and Mn^2+^. Further, the degradation of β-CY was almost constant in samples containing Zn^2+^ and Ca^2+^, indicating that these two metal ions did not significantly affect the co-metabolic degradation of β-CY.

**Table 3:** Degradation of β-CY by strain B-1 cells in suspension after the addition of metallic ions

Metallic ions	Concentrations (mmol/L)	β-CY degradation (%)
Control		22.19 ± 0.075^a^
Mg^2+^	0.1	26.14 ± 1.47^b^
	0.5	29.51 ± 2.19^b^
	1.0	22.58 ± 1.72^a^
	2.0	21.63 ± 1.42^a^
Mn^2+^	0.1	27.03 ± 0.92^b^
	0.5	28.98 ± 2.21^b^
	1.0	25.46 ± 1.43^b^
	2.0	22.59 ± 1.23^a^
Zn^2+^	0.1	22.70 ± 0.23^a^
	0.5	22.58 ± 1.41^a^
	1.0	21.09 ± 0.92^a^
	2.0	20.71 ± 2.31^a^
Ca^2+^	0.1	21.92 ± 2.12^a^
	0.5	21.79 ± 1.63^a^
	1.0	21.09 ± 1.12^a^
	2.0	20.12 ± 1.53^a^

Data are presented as mean ± standard deviation of three replicates; the standard deviations are within 5% of the mean. Different letters indicate significant differences (*p* < 0.05)

### Effects of ATP, NADH, and PEP on the co-metabolic degradation of β-CY

The effects of ATP and NADH on co-metabolic degradation of β-CY are presented in Table 4. β-CY degradation in samples with ATP were observed to be lower compared to those in the control, while β-CY degradation in samples with NADH were almost constant. Therefore, ATP could inhibit the activity of the degradation-associated enzyme of strainB-1.

**Table 4:** Degradation of β-CY by strain B-1 after the addition of NADH and ATP

Sample	Concentration (mmol/L)	β-CY degradation (%)
Control		23.42 ± 0.36^a^
Strain B-1 suspension with NADH	5.0	23.35 ± 0.28^a^
Strain B-1 suspension with ATP	5.0	19.85 ± 0.57^b^

Data are presented as mean ± standard deviation of three replicates; the standard deviations are within 5% of the mean. Different letters indicate significant differences (*p* < 0.05)

Co-metabolic degradation of β-CY after the addition of varying PEP concentrations are shown in Table 5. The degradation of β-CY was found to decrease with increasing PEP concentrations, indicating that PEP could inhibit the co-metabolic degradation of β-CY.

**Table 5:** Degradation of β-CY by strain B-1 after the addition of different concentrations of PEP

PEP concentrations (mmol/L)	β-CY degradation (%)
Control	22.68 ± 1.28^a^
5.0	22.09 ± 2.12^a^
10.0	17.01 ± 1.06^b^
15.0	14.52 ± 1.24^c^
20.0	12.31 ± 1.10^d^

Data are presented as mean ± standard deviation of three replicates; the standard deviations are within 5% of the mean. Different letters indicate significant differences (*p* < 0.05)

Taken together, the above findings showed that the β-CY degradation-associated enzyme was associated with the EMP. In particular, PEP inhibit the co-metabolic degradation. Furthermore, β-CY degradation could be promoted by the addition of Mg^2+^ and Mn^2+^, but can be inhibited by ATP, thereby suggesting that pyruvate kinase is the β-CY degradation-associated enzyme.

### Verification of the degradation-associated enzyme in strain B-1

To verified whether pyruvate kinase is the β-CY degradation-associated enzyme, the effects of activators and inhibitors of pyruvate kinase on the co-metabolic degradation of β-CY were investigated.

### Effects of pyruvate kinase activators on co-metabolic degradation of β-CY

**Fig. 2:**
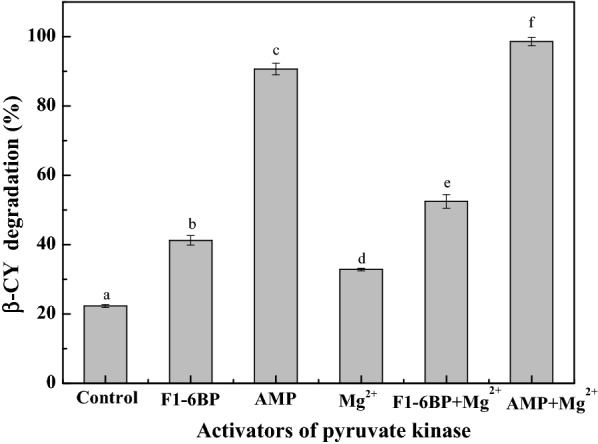
Effects of pyruvate kinase activators on co-metabolic degradation of β-CY by strain B-1. Data are presented as mean ± standard deviation of three replicates; the standard deviations are within 5% of the mean. Different letters indicate significant differences (*p* < 0.05)

Adenosine monophosphate (AMP), Mg^2+^ and F1-6BP were used as the pyruvate kinase activators (Tuominen and Bernlohr 1971). The effects of these activators on the co-metabolic degradation of β-CY by strain B-1 suspension are shown in Fig. 2. These activators were observed to improve β-CY degradation relative to the control. More specifically, β-CY degradation was found to be more efficient in samples containing AMP than those in samples containing F1-6BP or Mg^2+^. Furthermore, samples containing AMP and Mg^2+^ showed fivefold higher peak degradation than those in the control.

### Effects of pyruvate kinase inhibitors on the co-metabolic degradation

**Fig. 3:**
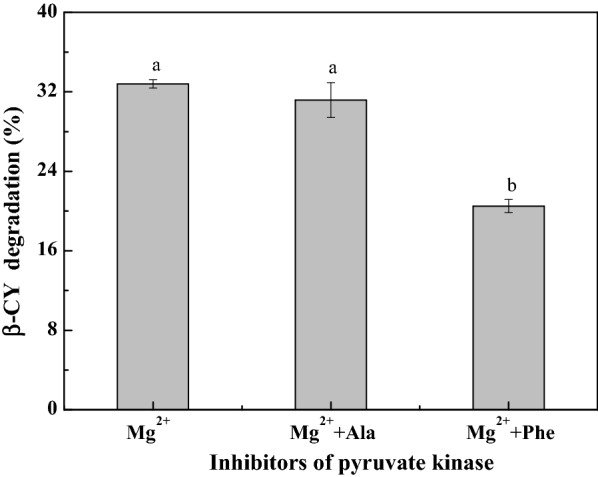
Effect of pyruvate kinase inhibitors on co-metabolic degradation of β-CY by strain B-1. Data are presented as mean ± standard deviation of three replicates; the standard deviations are within 5% of the mean. Different letters indicate significant differences (*p* < 0.05)

Alanine and Phe are generally used as inhibitors of pyruvate kinase (Tuominen and Bernlohr 1971; Zheng et al. 2014). The effects of Ala and Phe on the co-metabolic degradation of β-CY are shown in Fig. 3. Both of Ala and Phe could inhibit the degradation; however, samples with Phe showed higher degradation than that of samples containing Ala. The observed effects of activators and inhibitors of pyruvate kinase on the co-metabolic degradation of β-CY, evidently demonstrated that the activity of pyruvate kinase was positively associated with β-CY degradation by strain B-1.

### Nutrient regulation of co-metabolic degradation of β-CY by strain B-1 in corn flour

**Fig. 4:**
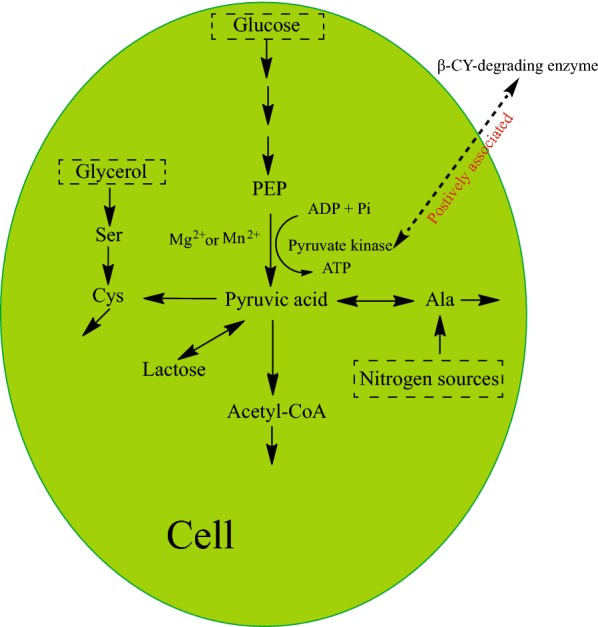
Proposed pathway for nutrient regulation during co-metabolic degradation of β-CY by strain B-1. Co-metabolic degradation of β-CY by strain B-1 was positively associated with the activity of pyruvate kinase, which could be regulated by glucose, glycerol, and nitrogen sources

The applications of corn in food were always restricted because of the presence of pesticide residues and its low protein content (Fields and Yoa 1990; Sharp et al. 1988). *Bacillus licheniformis* has been used to increase the protein content of corn flour (Fields and Yoa 1990). However, there is limited knowledge on β-CY degradation in corn flour. In this study, *Bacillus licheniformis* B-1 was used to degrade β-CY in corn flour, and the nutrient regulation during co-metabolic degradation of β-CY was verified by the addition of different nutrients which could regulate the activity of pyruvate kinase (Fig. 4).

### Effect of carbon sources on the co-metabolic degradation of β-CY

**Fig. 5:**
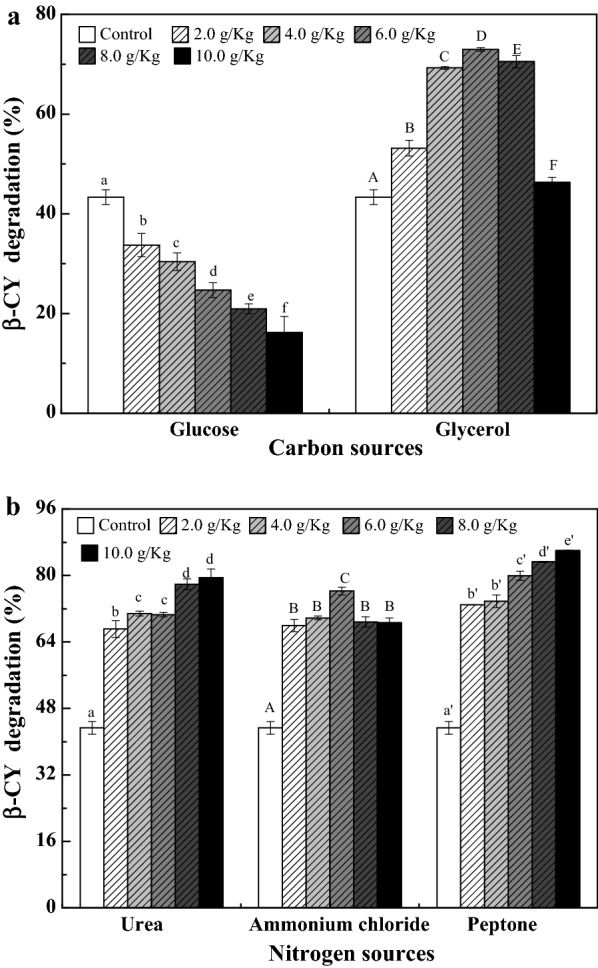
Effects of the addition of carbon (**a**) and nitrogen sources (**b**) on co-metabolic degradation of β-CY in corn flour by strain B-1. Data are presented as mean ± standard deviation of three replicates; the standard deviations are within 5% of the mean. Different letters indicate significant differences (*p* < 0.05)

The effects of various concentrations of glucose and glycerol on the co-metabolic degradation of β-CY are shown in Fig. 5a. β-CY degradation decreased with higher concentrations of glucose. The degradation of β-CY increased with increasing concentrations of glycerol at concentrations lower than 6.0 g/kg. By contrast, at glycerol concentrations ranging from 6.0 to 10.0 g/kg, the degradation decreased from 72.96 to 42.30%. These results suggested that the addition of optimal carbon sources in food could regulate and improve the β-CY degradation by microorganisms through co-metabolism.

### Effect of nitrogen sources on co-metabolic degradation of β-CY

The effects of urea, ammonium chloride and peptone on the co-metabolic degradation are shown in Fig. 5b. All three nitrogen sources were found to improve the co-metabolic degradation of β-CY by strain B-1 was observed to increase with higher concentrations of urea and peptone. At a concentration of 10 g/kg, the β-CY degradation peaked at 79.44% and 85.99% for urea and peptone, respectively. In addition, β-CY degradation was slightly higher when the ammonium chloride concentrations were lower than 6.0 g/kg, whereas opposite results were obtained at ammonium chloride concentrations higher than 6.0 g/kg. These results indicated that urea, peptone and ammonium chloride could improve the co-metabolic degradation of β-CY by strain B-1 in food.

## Discussion

In order to efficiently eliminate the pesticides residues in the food and environment, lots of microorganisms have been screened and isolated, and further studies mainly focused on the pathway for pesticides degradation, the characteristics of degrading-enzymes, and the cloning of degradation genes (Liu et al. 2014; Zhao et al. 2015, 2016, 2018). *Bacillus licheniformis* B-1 could not use β-CY as sole source of carbon (Additional file 1: Fig. S1), indicating it degraded β-CY via co-metabolism. In this work, we firstly investigated the substrate regulation on co-metabolic degradation of β-cypermethrin by strain B-1. One of the interesting finding was that the degradation of β-CY by strain B-1 did not consume energy. Previous studies reported similar findings, in which the degradation enzymes produced by *Aspergillus niger* ZD11 and *Sphingobacterium* sp. JZ-2 were demonstrated to be capable of degrading β-CY without requiring energy containing molecules (Guo et al. 2009; Liang et al. 2005). Sabullah et al. (2016) also reported that glyphosate could be degraded by the resting cells of *Klebsiella oxytoca*, demonstrating that glyphosate degradation did not consume energy. However, some reports indicated that co-metabolism by microorganisms requires energy for breaking chemical bonds in the stable chemical structures of organic contaminants (Luo et al. 2014; Nzila 2013; Tran et al. 2013). This is because energy dependence of the degradation of organic contaminants was dependent on the types of organic contaminants and microorganisms (Hatzinger et al. 2017; Ji et al. 2017). The explanation for above findings is that β-CY degradation by microorganisms occurs via hydrolysis, in which the ester bond is rarely involved in energy metabolism.

Enzymes required for co-metabolic degradation of organic contaminants simultaneously participate in the degradation and the metabolism of carbon source (Luo et al. 2014; Nzila 2013). It has been reported that strain B-1 degrade β-CY via co-metabolism (Liu et al. 2014; Zhao et al. 2015, 2016, 2018). Therefore, it can be speculated an association between the carbon metabolism of strain B-1 and the co-metabolic degradation of β-CY. The results indicated the degradation was correlated with EMP of strain B-1, because iodoacetic acid and sodium fluoride inhibit the activity of glyceraldehyde dehydrogenase phosphate and enolase the of EMP, respectively (Ostengo et al. 2005). Liu et al. (2013) reported that the co-metabolic degradation of imidacloprid was associated with hexose monophosphate pathway (HMP) in *Stenotrophomonas maltophilia*. Wang et al. (2019) indicated that the enzymes in TCA cycle of strain *Rhodococcus* sp. BAP-1 was related with degradation of fluoranthene by iTRAQ-based comparative proteomic analysis. Thereby, the compounds involved in EMP could affect the β-CY degradation.

The addition of certain divalent metal ions is known to increase the activity of some enzymes in the EMP (Calik and Ozdamar 1998; Voigt et al. 2004). The results indicated that higher concentrations of Mg^2+^ and Mn^2+^ impaired the biodegradation of β-CY, which was attributed to the inhibiting cell growth (Tang et al. 2011). While, low concentrations of Mg^2+^ and Mn^2+^ improved the β-CY degradation. Yi et al. (2012) reported that the activity of the pyrethroid-degrading enzyme in *Ochrobactrum anthropi* YZ-1 was increase by 17.60% when the Mg^2+^ and Mn^2+^ concentrations were set to 1.0 mmol/L. However, it is difficult to judge whether Mg^2+^ and Mn^2+^ promoted the activity of the degradation enzyme or the degradation-associated enzymes in strain B-1.

ATP and NADH are not only metabolites of the EMP, but additionally provide energy or serve as cofactors for co-metabolic enzymes (Calik and Ozdamar 1998; Voigt et al. 2004). Lu et al. (2018) reported that adding ATP and NADH could enhance the transformation of organic component. Our results indicated that ATP inhibited the β-CY degradation, while NADH did not significantly affect it. However, previous studies reported contradictory results, in which ATP or NADH were found to improve the degradation of organic contaminants by microorganisms (Luo et al. 2014; Nzila 2013; Tran et al. 2013) and was attributed to differences in the chemical structures of organic contaminants containing pyrethroid (Luo et al. 2014). Moderate amounts of ATP are not toxic to microorganisms. Although enzymatic inhibition by ATP was unexpected, a possible explanation was that the co-metabolic degrading enzyme of β-CY or the degradation-associated enzyme could utilize growth substances to produce ATP, which in turn inhibited enzymatic activity through feedback inhibition.

Above results indicated that the enzyme responsible for β-CY degradation in strain B-1 did not produce and consume ATP during co-metabolic degradation of β-CY. Therefore, ATP could inhibit the activity of the degradation-associated enzyme of strainB-1. Moreover, AMP was speculated to promote β-CY degradation by strain B-1, which was validated in Fig. 2.

As an important metabolite of the EMP, phosphoenolpyruvate (PEP) could be metabolized by pyruvate kinase to produce ATP in the presence of Mg^2+^ and Mn^2+^ (Calik and Ozdamar 1998; Voigt et al. 2004). PEP showed negative effects on β-CY degradation. There are two possible explanations for the above results. First, PEP could act as the substrate of the β-CY degradation-associated enzyme, and the product (ATP) would inhibit the activity of the enzyme. Second, PEP could be the product of the β-CY degradation-associated enzyme, and can inhibit β-CY degradation via feedback inhibition.

The allosteric effect of AMP on the pyruvate kinase of *Bacillus licheniformis* is stronger than that of F1-6BP (Tanaka et al. 1995). Pyruvate kinase activators have different mechanisms of action (Tanaka et al. 1995; Tuominen and Bernlohr 1971; Zheng et al. 2014), resulting β-CY degradation in the samples containing F1-6BP, Mg^2+^, F1-6BP and Mg^2+^ were 41.24%, 32.81% and 52.47%, respectively. Meanwhile, the inhibiting effects of Ala and Phe were also different, which is attributed to the allosteric effect of Phe than that of Ala (Muñoz and Ponce 2003). Ala binds to the deep pocket of pyruvate kinase located between the A and C domains, which is distant from the active site; the binging of Phe to pyruvate kinase leads to higher flexibility of the protein structure (Muñoz and Ponce 2003; Williams et al. 2006; Zheng et al. 2014).

Favaloro et al. (2000) reported that glutathione synthase was degradation-associated enzyme for the co-metabolic degradation of atrazine by *Ochrobactrum anthropic*, and its regulatory factors could affect atrazine degradation. For microorganisms via co-metabolism, the only way to regulate the efficiency of microbial degradation of organic pollutants present in foods was to investigate the characteristics of degrading enzyme or degradation-associated enzyme (Liu et al. 2013).

Taken together, previous findings and the current results indicated that β-CY degradation could be regulated by controlling the activity of pyruvate kinase, which was strongly influenced by the presence of specific nutrients. Based on previous finding on the metabolic pathways in *Bacillus licheniformis* (Voigt et al. 2004), the regulatory pathway for β-CY degradation is summarized in Fig. 4. β-CY degradation was positively associated with the activity of pyruvate kinase, the key enzyme involved in the EMP (Schettgen et al. 2002). It is well known that carbon and nitrogen sources that are associated with the EMP pathway can influence pyruvate kinase activity (Muñoz and Ponce 2003; Tuominen and Bernlohr 1971; Voigt et al. 2004; Zheng et al. 2014). Thus, the co-metabolic degradation of β-CY could be improved by the addition of nutrients that increase the activity of pyruvate kinase. As shown in Fig. 4, glucose, glycerol and nitrogen sources could effectively regulate the activity of pyruvate kinase.

In corn flour, the concentrations of glucose were negatively associated with β-CY degradation, because the glucose could be converted into PEP through the EMP (Voigt et al. 2004) and high concentrations of PEP would inhibit β-CY degradation (Table 5). Meanwhile, high concentrations of glycerol also could reduce the degradation of β-CY; while, at low concentrations, it could improve the degradation. It is likely that *Bacillus licheniformis* preferentially utilizes glycerol than starch (Voigt et al. 2004) and the activity of pyruvate kinase can be enhanced at low glycerol concentrations. However, at higher glycerol concentrations, high concentrations of cysteine produced during glycerol metabolism inhibit enzymatic activity of pyruvate kinase. Similar results were reported by Liu et al. (2013) in sample containing sucrose, glucose, or fructose, which were observed to have significantly higher imidacloprid degradation. The results indicated that the degradation of β-CY was increased as the concentrations of urea and peptone increased. These findings are attributed to the relatively low nitrogen content in corn flour, and the addition of urea and peptone provided nitrogen sources for the growth of strain B-1 (Calik and Ozdamar 1998; Fields and Yoa 1990; Voigt et al. 2004). Although ammonium chloride also could improve the degradation, when the concentrations higher than 0.6 g/kg the degradation decreased. It is likely that ammonium chloride could improve the growth of strain B-1 at lower concentrations; however, at higher ammonium chloride concentrations, large amounts of hydroxylamine can be produced by microbial nitrification and converted to phosphoramide via a hydroxylamine reaction, which in turn inhibits the growth of strain B-1 (Nowak and Suelter 1981). These results demonstrated the degradation of β-CY could be improved by regulating the activity of pyruvate kinase.

## Additional file

**Additional file 1: Fig. S1.** Concentrations of β-CY in MS-CY medium for 72 h by strain B-1.

## Data Availability

The dataset supporting the conclusions of this article is included within the article. All data are fully available without restriction.

## Abbreviations

β-CY: β-cypermethrin
NADH: nicotinamide adenine dinucleotide
ATP: adenosine triphosphate
GTP: guanosine triphosphate
AMP: adenosine monophosphate
PEP: phosphoenolpyruvate
F1-6BP: fructose 1,6 bisphosphate
Ala: alanine
Phe: phenylalanine
HPLC: high-performance liquid chromatograph
EMP: Embden–Meyerhof–Parnas
HMP: hexose monophosphate pathway
TCA cycle: tricarboxylic acid cycle
