# Supplementary material for: Substrate regulation on co-metabolic degradation of β-cypermethrin by Bacillus licheniformis B-1
Source: AMB Express. 2019 Jun 12;9:83. doi: 10.1186/s13568-019-0808-3 (PMC6562013; doi:10.1186/s13568-019-0808-3)
Supplement: Supplementary file 1 — Additional file 1: Fig. S1. Concentrations of β-CY in MS-CY medium for 72 h by strain B-1. [file 13568_2019_808_MOESM1_ESM.docx]

**Fig. S1**





**Concentrations of β-CY in MS-CY medium for72 h by strain B-1**

Mineral salt (MS) medium containing 0.2 g/L MgSO_4_, 0.5 g/L KH_2_PO_4_, 0.5 g/L NaCl, 1.5 g/L (NH_4_)_2_SO_4_, and 1.5 g/L K_2_HPO_4_ was prepared. MS-CY medium consisted of MS medium and β-CY (100 mg/L). The pH value of each medium was adjusted to 7.0-7.5, and 0.2% (v/v) of ethyl alcohol was added into the medium as a hydrotropic agent before sterilization at 121ºC for 20 min.
